# Association of the chemical composition and nutritional value of forage resources in Colombia with methane emissions by enteric fermentation

**DOI:** 10.1007/s11250-023-03458-x

**Published:** 2023-02-16

**Authors:** Yiniva Camargo Caicedo, Angélica P. Garrido Galindo, Inés Meriño Fuentes, Eliana Vergara Vásquez

**Affiliations:** Research Group On Environmental Systems Modelling, Universidad del Magdalena, Carrera 32 N° 22 – 08, Postal Code 470004 Santa Marta, Colombia

**Keywords:** Mitigation strategies, ARIMA, Livestock diet, Climate change, Livestock diet composition, Gramineous specie

## Abstract

In the livestock sector, strategies are available to mitigate gas emissions, such as methane, one of the alternatives that have shown potential correspondence to changes in the composition of the diet. The main aim of this study was to analyze the influence of methane emissions with data on enteric fermentation obtained from the Electronic Data Gathering, Analysis, and Retrieval (EDGAR) database and based on forecasts of methane emissions by enteric fermentation with an autoregressive integrated moving average (ARIMA) model and the application of statistical tests to identify the association between methane emissions from enteric fermentation and the variables of the chemical composition and nutritional value of forage resources in Colombia. The results reported positive correlations between methane emissions and the variables ash content, ethereal extract, neutral detergent fiber (NDF), and acid detergent fiber (ADF) and negative correlations between methane emissions and the variables percentage of unstructured carbohydrates, total digestible nutrients (TDN), digestibility of dry matter, metabolizable energy (MERuminants), net maintenance energy (NEm), net energy gain (NEg), and net lactation energy (NEI). The variables with the most significant influence on the reduction of methane emissions by enteric fermentation are the percentage of unstructured carbohydrates and the percentage of starch. In conclusion, the analysis of variance and the correlations between the chemical composition and the nutritive value of forage resources in Colombia help to understand the influence of diet variables on methane emissions of a particular family and with it in the application of strategies of mitigation.

## Introduction

The nitrous oxide and methane emitted by ruminants constitute greenhouse gases with a significant impact on the environment and a significant influence on the sustainability of production systems in this sector (Jaurena et al., 2019; Tigmasa Paredes, 2022).

Methane emissions in terms of energy constitute a loss (Bai et al., 2021), and in environmental terms, they contribute to global warming and consequently to climate change (Tigmasa Paredes, 2022). The effects occur directly through their interaction with infrared energy and indirectly through atmospheric oxidation reactions (Carmona et al., 2005). About 17% of the gross energy consumed by a cow is transformed into methane and is eliminated through the respiratory tract (Tigmasa Paredes, 2022).

In the livestock sector, the enteric fermentation and microbial degradation of feces make essential contributions to methane and nitrous oxide emissions (Huhtanen et al., 2021). Ruminant animals, such as cattle, buffalo, sheep, goats, and camels, produce methane from digestive processes that occur under anaerobic conditions through the action of microbiota such as methanogenic archaea that live in the rumen and use carbon dioxide (CO_2_) and hydrogen (H_2_) to form methane (CH_4_) (Wang et al., 2022)_._ In the digestive process of cattle under anaerobic conditions, bacteria degrade ingested cellulose to glucose. Through the fermentation process, these bacteria transform cellulose into acetic acid and reduce carbon dioxide, generating methane during the process (Carmona et al., 2005).

Methane production in ruminants is influenced by different factors, such as diet composition, feed consumption, previous feed processing, nutrient digestibility, and feeding frequency (Dai et al., 2022; Ribeiro da Silva et al., 2020).

Livestock production contributes to anthropogenic emissions of carbon dioxide (CO_2_), methane (CH_4_), and nitrous oxide (N_2_O) to the atmosphere (Costantini et al., 2018; Ribeiro da Silva et al., 2020; Tigmasa Paredes, 2022). Methane is emitted in smaller quantities; however, it has a much higher global warming potential (Tigmasa Paredes, 2022), 28 times that of CO_2_ for a horizon of 100 years, according to an Intergovernmental Panel on Climate Change (IPCC) report in 2014 (IPCC-Grupo Intergubernamental de expertos sobre el cambio climático, 2014).

It is estimated that the livestock sector worldwide is responsible for 17% of methane (Carrillo-Hernández et al., 2021; Pámanes-Carrasco et al., 2020). Latin American countries are characterized by low production of meat and milk of animal origin. They have a high production of methane emissions, about 69%. In this context, the tendency is marked to own many unproductive animals instead of keeping a few animals with high production (Yunga Alava, 2022).

According to a report by Ribeiro da Silva et al. (2020), in Latin America, on average, a bovine emits 56 kg/animal/year of enteric methane. In this context, the production of beef generates emissions with values close to 300 kg CO_2_ eq/kg protein, followed by the production of meat from small ruminants, with a value of 165 kg CO_2_ eq/kg protein, and milk production by small ruminants, with a value of 112 kg CO_2_ eq/kg protein (Durango et al., 2017).

Different alternatives have been proposed as mitigation strategies for methane emissions, such as reducing the number of ruminant animals, increasing the number of nonruminant animals, genetic manipulation of methanogenic ruminal microorganisms, dietary-nutritional manipulation, capture mechanisms for critical compounds, and supplementation practices with nitrogen sources (Alayón-Gamboa et al., 2018; Liu et al., 2022; Ribeiro da Silva et al., 2020; Tigmasa Paredes, 2022).

According to different publications, the diet influences greenhouse gas emissions. The rate of ruminal emissions is related to the composition of the diet, types of carbohydrates, proteins, and lipid value (Arango et al., 2016; Wang et al., 2022). The results of advanced studies suggest that an increase in lipids in diets can reduce methane emissions in ruminants (Dai et al., 2022), in the same way, forages with high starch content in the diet of ruminants reduce the production of enteric methane (Wang et al., 2022).

Concerning to an increase in rumen undegradable protein, analyses performed show a reduction in methane production per kg of digested organic matter (Ribeiro da Silva et al., 2020). On the other hand, an increase in complex carbohydrates in the diet contributes to methane production (Kumari et al., 2020). In general, the reduction of enteric methane emissions is related to the quality of the diet and the digestibility of nutrients (Dai et al., 2022).

On the other hand, the time series forecasts of emissions are essential tools for estimating data that allow future decision-making. In the livestock sector, applying quantitative models based on historical data has proven to be helpful in the realization of time series forecasts applied to emissions of greenhouse gases such as methane (Sutthichaimethee & Ariyasajjakorn, 2017; Yusuf et al., 2014).

Taking into account the different conclusions of publications regarding the influence of the diet of the livestock sector on methane emissions, we worked on the next hypothesis: the behavior of the variables of the chemical composition and nutritional value of the forage resources of Colombia’s grass family has a different correlation with the behavior of methane emissions due to enteric fermentation in Colombia.

The main objective of this study is to determine the influence of the chemical composition and nutritional value of forage resources in Colombia for the grass family on methane emissions by enteric fermentation. This is based on forecasts of methane emissions by enteric fermentation and information on the chemical composition and nutritional value provided by the Colombian Agricultural Research Corporation. In accordance with the results, contribute to the generation of information on the application of practices that favor the reduction of greenhouse gas emissions in the livestock sector and that help meet the goal of reducing greenhouse gases in Colombia by 2030 within the framework of the contribution at the national level in Colombia–NDC.

## Materials and methods

### General

The methodology used was quantitative and descriptive, with Colombia as the study area. Considering the studies on the estimation of greenhouse gases (Cheewaphongphan et al., 2019; Parker et al., 2018; Peng et al., 2016; Saunois et al., 2020), emission data provided by the Emissions Database for Global Atmospheric Research–Electronic Data Gathering, Analysis and Retrieval (EDGAR) v5.0 were used. Based on the methodology developed by Cujia et al. (2019), Nyoni and Mutongi (2019), Sutthichaimethee and Ariyasajjakorn (2017), and Yusuf et al. (2014), a forecast of methane emissions from enteric fermentation was obtained with an autoregressive integrated moving average (ARIMA) model to determine the correlations with the chemical composition and nutritional value of the forage resources of Colombia. This model was used because there were no methane emissions by enteric fermentation from the years 2016–2020 in the EDGAR v5.0 database. The chemical composition and nutritional value of the forage resources were obtained from the open database of Colombia provided by the Colombian Agricultural Research Corporation AGROSAVIA. The statistical software used for data analysis was RStudio.

### Identification of emissions and data on chemical composition and nutritional value

#### Database review

The data used in the research were obtained from databases. The records of CH_4_ emissions by enteric fermentation from 1992–2015 were downloaded from the Emissions Database for Global Atmospheric Research—EDGAR v5.0. The records of the chemical composition and nutritional value of forage resources in Colombia for the years 2013–2020 were obtained from an open database of Colombia. The open database contains data provided by the Colombian Agricultural Research Corporation AGROSAVIA; the information are derived from the system of food information from the tropics for animal feed—AlimenTro.

#### Forecast of methane emissions by enteric fermentation

According to the methodology of Yusuf et al. (2014); Sutthichaimethee and Ariyasajjakorn (2017); Cujia et al. (2019); and Nyoni and Mutongi (2019), to forecast methane emissions by enteric fermentation for 2016–2020, historical data were taken from the EDGAR database from 1992 to 2015, and the “ARIMA (p, d, q),” where *p* is the number of parameters of the autoregressive process (AR), *d* is the number of parameters of the differentiation process (I), and *q* is the number of parameters for the moving average (MA) process, was used.

For the application of the ARIMA model, the stationarity of the series was verified. The series was not stationary, so a natural logarithm was applied to the data. Then, the stationarity of the mean was verified. Then, the autocorrelation was verified to review the correlation of the lags. Finally, the model was applied in the statistical software R with the values *p* = 3, *d* = 0, and *q* = 0 according to the verifications performed before the application of the model.

The assumptions of independence, white noise, and normality of the residuals were verified.

Subsequently, the data were transformed to their original form, and the complete series was obtained with forecasts up to 2020; these were used to analyze methane emissions with the chemical composition and nutritional value of the averaged data from Colombia.

#### Data processing

##### Methane emissions by enteric fermentation.

The conversion of methane emissions (CH4) from Colombia, obtained from the EDGAR v5.0 database, into equivalent CO_2_ emissions was carried out considering the methodological guidelines of the IPCC in 2006 and according to the National Inventory and departmental of greenhouse gases–Colombia of the Institute of Hydrology, Meteorology and Environmental Studies, Program for the United Nations for the development, Ministry of Environment and Sustainable Development, National Department of Planning and Chancellery.

According to IDEAM et al. (2016), “To convert the mass of each GHG into CO_2_ equivalent, the Global Warming Potential (GWP) is used, which is a relative value that expresses how much-infrared radiation GHG traps in the atmosphere relative to the trapped by CO_2_ in different time horizons (20, 100 and 500 years). For example, the GWP of CH_4_ reported by the IPCC in its second assessment report for a time horizon of 100 years is 21, which means that 1 kg of CH_4_ traps 21 times more infrared radiation than 1 kg of CO_2_. This means, that climatically 1 kg of CH_4_ is equivalent to 21 kg of CO_2_ equivalent.”

Therefore, GWPs from the IPCC Climate Change Assessment reports were used. For the emissions of the years 1992–1994, a GWP of 11 was applied; for the emissions of the years 1995–2000, a GWP of 21; 2001–2006 a GWP of 23 was applied; 2007–2013 a global warming potential of 25; and for the emissions of the years from 2014, a global warming potential of 28 was used (IPCC-Grupo Intergubernamental de expertos sobre el cambio climático, 2014; IPCC-Intergovernmental Panel on Climate Change, 1992, 1996, 2001, 2007).

Based on the above, the concentration data expressed in gigagrams of CH_4_ from the EDGAR v5.0 database and the forecast from the ARIMA model were multiplied by the corresponding GWP of methane for each year, and methane emissions by enteric fermentation expressed in kg CO_2_ eq for 1992–2020 were obtained.

##### Chemical composition and nutritional value of forage in Colombia.

Averages of the samples of the variables downloaded from the open database were calculated. Due to the availability of data, gramineous species were selected, and an average was determined to obtain a national average of the analyzed variables of crude protein, ash content, ethereal extract, neutral detergent fiber (NDF), acid detergent fiber (ADF), hemicellulose, lignin, percentage of total starch, percentage of soluble carbohydrates, percentage of nonstructural carbohydrates, total digestible nutrients (TDN), dry matter digestibility, metabolizable energy (MERuminants), net maintenance energy (NMERuminants), net energy gain (NEGRuminants), and net lactation energy (NLERuminants).

### Association of emissions with data on chemical composition and nutritional value of forage resources

With the methane emissions data by enteric fermentation obtained from the EDGAR v5.0 database and the emissions forecast with the ARIMA model, the association of the emissions from 2013 to 2020 with the chemical composition and nutritional value of the forage resources in Colombia was verified. For the comparison, the following variables were taken into account: crude protein, ash content, ethereal extract, NDF, ADF, hemicellulose, lignin, percentage of total starch, percentage of soluble carbohydrates, percentage of non-structural carbohydrates, TDN, dry matter digestibility, MERuminants, NMERuminants, NEGRuminants, and NLERuminants.

Analysis of variance (ANOVA) was applied to verify significant differences statistically, and a correlation test was applied in the software RStudio to determine the association between the variables of chemical composition, nutritional value, and CH_4_ emissions by enteric fermentation.

## Results

### Methane emissions forecast

**Fig. 1:**
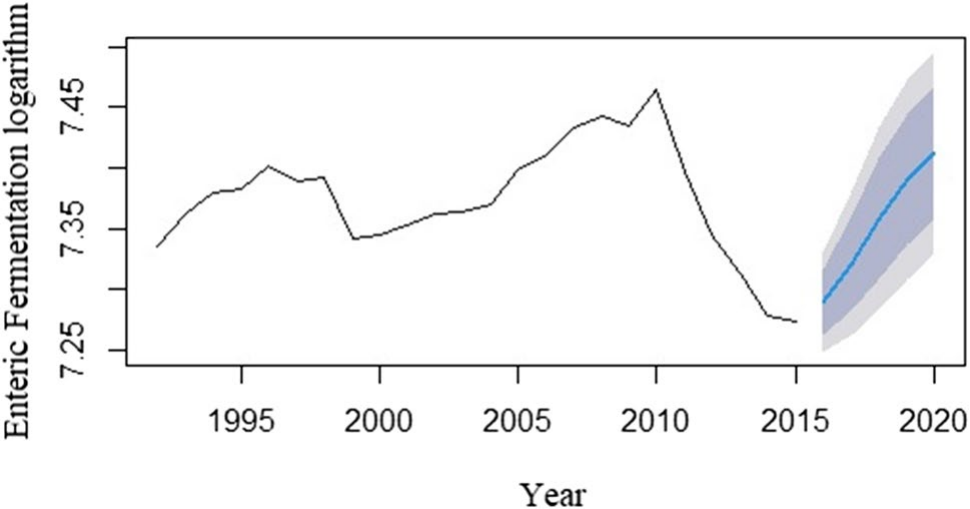
Forecast of the transformed time series with the ARIMA model

Figure 1 presents the result of the forecast of the transformed series for the data of methane emissions by enteric fermentation with the ARIMA model using model (3,0,0). The black line for 1992–2015 corresponds to the series adjusted by the model. The gray band corresponds to the prediction interval with a confidence level of 95%. In Table 1, the validation results of the assumptions of independence, white noise, and normality of the residuals are reported; according to the records in the model, the three assumptions are met.

**Table 1:** Validation of the ARIMA model assumptions

	Box-Ljung Test			Jarque Bera Test		
	*X* square	df	*P* value	*X* square	df	*P* value
Methane emissions by enteric fermentation	0.48356	1	0.4868	0.062854	2	0.9691

### Processing of methane emissions

**Fig. 2:**
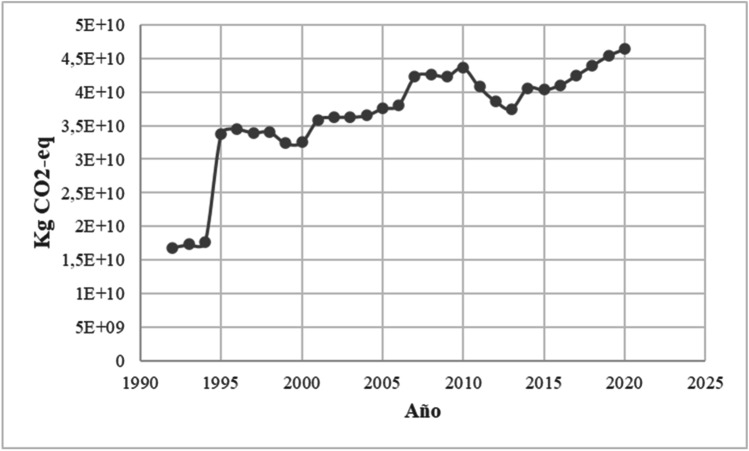
Methane emissions by enteric fermentation from 1992–2020

In Fig. 2, the results of the processing of methane emissions by enteric fermentation from 1992–2020 are presented, including the results of methane emissions obtained from the forecast for the years 2016–2020 from the ARIMA model. An increasing trend is observed for the years 2016–2020 compared to the years 1992–2015, which corresponds to the results of the emissions processed with the IPCC methodology in 2006 from the EDGAR v5.0 database. The minimum value occurred in 1992, with a value of 16,869,061,000 kg CO_2_ eq, and the maximum value, 46,404,624,000 kg CO_2_ eq, occurred in 2020.

### Association of emissions with data on chemical composition and nutritional value of forage resources

#### Normality test

The results of the Shapiro–Wilks normality test applied to data from 2013–2020 (Table 2) report *P* values greater than 5% for the records of the variables of chemical composition, nutritional value, and methane emissions by enteric fermentation, which shows that the data follow a statistically normal distribution. Based on the normal behavior of the data, ANOVA is applied in the statistical software RStudio.

**Table 2:** Test of the normality variables of methane emissions, chemical composition, and nutritional value from 2013–2020

Chemical composition and nutritional value variables 2013–2020			
	Statistic (W)	*P* value	Significance
Crude protein	0.88777	0.2231	Normal
Ash content	0.92524	0.4738	Normal
Ether extract	0.90956	0.3509	Normal
NDF	0.93542	0.5666	Normal
ADF	0.90465	0.3179	Normal
Hemicellulose	0.97168	0.9108	Normal
Lignin	0.87148	0.1558	Normal
Percentage total starch	0.72716	0.005	Normal
Percentage soluble carbohydrates	0.91782	0.4124	Normal
Nonstructural carbon	0.8897	0.2329	Normal
TDN	0.92874	0.5047	Normal
Digestibility of dry matter	0.92862	0.5036	Normal
EDRuminants	0.93323	0.5459	Normal
MERuminants	0.92869	0.5043	Normal
NMERuminants	0.92519	0.4734	Normal
NEGRuminants	0.92305	0.4551	Normal
NLERuminants	0.91322	0.3773	Normal
Methane emissions 2013–2020			
CH_4_ enteric fermentation	0.96773	0.8796	Normal

#### ANOVA test

The ANOVA test reports a *P* value < 2e-16, which indicates that the significance is less than 5%; therefore, the null hypothesis that there are no statistically significant differences between the variables analyzed is rejected (Table 3).

**Table 3:** ANOVA test

Variance analysis–methane emissions and total chemical composition and nutritional value—RStudio					
			Group	Residuals	
	Sum of squares		1.35E + 22	6.04E + 19	
	Degrees of freedom		17	126	
	Residual standard error		6.92E + 08		
	GL	Sum of squares	Quadratic mean	F value	Pr (> F)
Group		1.35E + 22	7.92E + 20	1653	< 2e-16
Residuals		6.04E + 19	4.79E + 17		

#### Correlation of emissions and chemical composition and nutritional value data

The variables of the chemical composition and nutritional value (Table 4) like ash content, ethereal extract, NDF, lignin, and ADF show positive correlations, while the variables raw protein, hemicellulose, percentage starch total, percentage of non-structural carbohydrates, TDN, digestibility of dry matter, ED, ME, NEm, NEg, and NEI show negative correlations with methane emissions.

**Table 4:** Correlation test of methane emissions and data on the chemical composition and nutritional value of the diet

	CH_4_ emissions from enteric fermentation
Raw protein	− 0.47571523
Ash content	0.75593614
Ethereal extract	0.4298877
NDF	0.4501952
ADF	0.5961028
Hemicellulose	− 0.007136338
Lignin	0.4068881
Percentage starch total	− 0.72937543
Percentage soluble carbohydrates	− 0.26240434
Percentage nonstructural carbon	− 0.7641809
TDN	− 0.6813214
Digestibility of dry matter	− 0.6812370
EDRuminants	− 0.6405773
MERuminants	− 0.6813367
NMERuminants	− 0.6778772
NEGRuminants	− 0.6769901
NLERuminants	− 0.6984954

## Discussion

The results of the methane emissions forecast by enteric fermentation obtained in Fig. 1 are consistent with the trend of methane emissions estimated for the years 1992–2015 from the IPCC methodology in 2006, the same as the studies published by Nyoni and Mutongi (2019), Sutthichaimethee and Ariyasajjakorn (2017), and Yusuf et al. (2014). The application of the ARIMA model is adequate and stable for the estimation of greenhouse gas emissions because the results of the model shown in Table 1 indicate the fulfillment of the assumptions of independence, white noise, and the normality of the residuals.

The results of the ANOVA test (Table 3) show that there are statistically significant differences between the data on methane emissions by enteric fermentation and the analyzed variables of chemical composition and nutritional value of forage resources for the grass category in Colombia. This result allows us to infer that the composition of the diet is a significant factor influencing methane emissions from enteric fermentation.

The above coincides with the results of studies published by Arango et al. (2016), Hristov et al. (2013), Kumari et al. (2020), and Soto (2015), where methane emissions from the livestock sector were constantly associated with the composition of the diet and the intervention of different variables that compose it, such as carbohydrates, protein value, percentage of fat, starch, digestibility, and quality of the diet.

Consequently, the correlations between methane emissions by enteric fermentation and the variables of chemical composition and nutritional value of forage resources for the grass category in Colombia (Table 4) show that increases in the variables of the percentage of non-structural carbohydrates, percentage of total starch, TDN, digestibility of dry matter, ME, NEm, NEg, and NEI, contribute to the reduction of methane emissions. Authors such as Alayón-Gamboa et al. (2018), Arango et al. (2016), Hristov et al. (2013), Nuñez-Hernández et al. (2015), Ungerfeld et al. (2018), and Valencia-Trujillo and Rojas-López (2019) show this behavior in their results.

The literature shows that the content of dietary elements such as lipid, protein, and carbohydrate content can reduce methane emissions from enteric fermentation. However, according to the study’s results, these elements may vary depending on the family analyzed.

In the case of the Colombian data, it was observed that in the grass family, the elements with the highest negative correlations are the percentage of total starch and the percentage of non-structural carbohydrates, which suggests that more significant reductions in methane emissions would be obtained with this species if the content of starch and non-structural carbohydrates are increased. Although protein content showed a negative correlation, it did not show as high a negative correlation as starch content. In the case of the grass species, the rapid fermentation of starch decreases the ruminal pH, favoring the formation of propionate, which contributes to reducing the availability of hydrogen for the formation of methane, following what was stated by Carrillo-Hernández et al. (2021).

This result may be because the grass family is characterized by its low protein content; in fact, the author Herranz (2018) points out that grasses contain half the crude protein compared to legumes, which can explain the negative correlation but less intense than the correlation with the percentage of starch and non-structural carbohydrates. Similarly, the high negative correlation between the percentage of starch and non-structural carbohydrates may indicate that they are young plants, since in mature forages, the content of structural carbohydrates is increased, and the content of more fermentable carbohydrates is reduced, resulting in higher methane production.

In the same way, it is clarified that the reduction of methane emissions can be linked, in addition to the presence of protein, to the type of protein; according to Ribeiro da Silva et al. (2020), the type of non-degradable protein in the rumen can limit the growth of methanogenic microorganisms and thereby reduce the total population of methane-producing archaea in the rumen, due to the low availability of the H and methyl group in the rumen.

However, according to the results of Ribeiro da Silva et al. (2020), more studies should be carried out to directly associate the reduction in methane emissions, with the decrease in the population of methane-producing archaea, associated with the content of non-degradable protein.

On the other hand, positive correlations with higher values occur for the variables, ash content, ethereal extract, NDF, and ADF, which indicates that increases in these variables imply more significant emissions of methane; the above coincides with that reported by Arango et al. (2016), Hristov et al. (2013), and Valencia-Trujillo and Rojas-López (2019).

However, the results regarding the influence of the variables on methane emissions from the livestock sector vary. In this sense, in addition to the variables, the quantities of each variable also sometimes have an influence, as mentioned in Hristov et al. (2013); a decrease in a variable can increase in another variable, and therefore, the amounts of each component in the diet must be in proportions that allow maintaining the nutritional balance and at the same time the reduction of emissions.

The results are based on the averages of the chemical composition and nutritional value of the forage resources in Colombia of several departments, so the number of departments is not constant during all the years evaluated from 2013–2020, which may influence the results discussed.

Finally, as a conclusion of the study, the following are mentioned:

The forecast of methane emissions by enteric fermentation with the ARIMA model, it can represent a helpful tool for making predictions in the livestock sector in Colombia, in the same way, the analysis of variance and the correlations between the chemical composition and nutritional value of the forage resources in Colombia show to be viable statistical tools to understand the specific influence of the diet variables on the methane emissions of a particular family and thus help to the projection of strategies for the reduction of methane emissions in the Colombian livestock sector.

## Data Availability

The datasets generated and analyzed during the current study are available from the corresponding author on reasonable request.
